# From Radiological Manifestations to Pulmonary Pathogenesis of COVID-19: A Bench to Bedside Review

**DOI:** 10.1155/2020/8825761

**Published:** 2020-12-04

**Authors:** Amin Saburi, U. Joseph Schoepf, Kyle A. Ulversoy, Ramezan Jafari, Fatemeh Eghbal, Mostafa Ghanei

**Affiliations:** ^1^Chemical Injuries Research Center, Systems Biology & Poisonings Institute, Baqiyatallah University of Medical Sciences, Tehran, Iran; ^2^Department of Radiology and Radiological Science, Medical University of South Carolina, Charleston, SC, USA; ^3^Augusta University/University of Georgia Medical Partnership, Athens, GA, USA; ^4^Birjand University of Medical Sciences, Birjand, Iran

## Abstract

In this review, we aim to assess previous radiologic studies in COVID-19 and suggest a pulmonary pathogenesis based on radiologic findings. Although radiologic features are not specific and there is heterogeneity in symptoms and radiologic and clinical manifestation, we suggest that the dominant pattern of computed tomography is consistent with limited pneumonia, followed by interstitial pneumonitis and organizing pneumonia.

## 1. Introduction

There are many studies on the radiologic manifestations of pulmonary involvement of novel coronavirus disease (named COVID-19 by the WHO) first reported from Wuhan, China. High-resolution computed tomography (HRCT) of the lung and chest X-ray are the most frequent and valuable studies to be used, but nuclear medicine scans and catheter angiography have also been used [1].

There are many HRCT findings frequently reported, including ground-glass opacity (GGO), interseptal thickening with or without the nodular pattern, subpleural sparing, and prominent vasculature. However, extensive tiny lung nodules, lymphadenopathy, pleural effusion, and pleural involvement are less frequently reported. If the previously stated findings are seen, bacterial or non-COVID-19 infections should also be kept in mind [2–4].

Almost all previous studies are focused on clinical and radiological manifestations of COVID-19, but main pulmonary pathology that explains these manifestations is less extensively discussed. It is possible to find or at least infer main pathologies based on radiologic manifestations as previously reported for similar diseases such as severe acute respiratory syndrome (SARS-CoV) or Middle East respiratory syndrome (MERS) [5].

In this review, we aim to assess previous radiologic studies on COVID-19 and to suggest pulmonary pathogenetic pathways based on radiologic findings.

## 2. Review of Clinical Findings

There are many reports about clinical features and epidemiologic characteristics of patients affected with COVID-19. These findings are sometimes contradictory, which may be due to population heterogeneity, differences in sample size, and selection bias. Likewise, racial and environmental characteristics in the published studies can show different results.

In one of the largest studies published in the New England Journal of Medicine, the following findings were obtained: of 1099 enrolled subjects, more than 41% were female and the mean incubation time was 4 days, less than 1% were under 15 years old (pediatric cases), and the two most frequent symptoms on admission were fever and cough. About 15% of admitted cases had severe symptoms. They reported that “the presence of any coexisting illness was more common among patients with severe disease than among those with nonsevere disease (38.7% vs. 21%).” The median age of admitted cases was 47 (35–58) y/o [6].

These findings are similar to other studies such as PingZhengMo et al.'s study who reported 155 cases with COVID-19 pneumonia. They described that the median age was 54 y/o and “Fever (81.3%), fatigue (73.2%), cough (62.6%), and myalgia/arthralgia (61.0%) were the most common symptoms” [7]. Acute respiratory distress syndrome (ARDS) was the most common indication for ICU admission in COVID-19 cases and is also the major etiology of death [8]. Age > 50 years, dyspnea, comorbidities, chest pain, cough, expectoration, increased serum inflammatory factors, and decreased lymphocytes count, specially obesity (visceral fat) were reported as risk factors for the critical phase [9, 10]. Moreover, milder and less prominent clinical symptoms were reported in pediatric patients, with a recovery period of 1-2 weeks, which is shorter than in adult cases [11].

## 3. Review of Radiologic Findings

**Figure 1:**
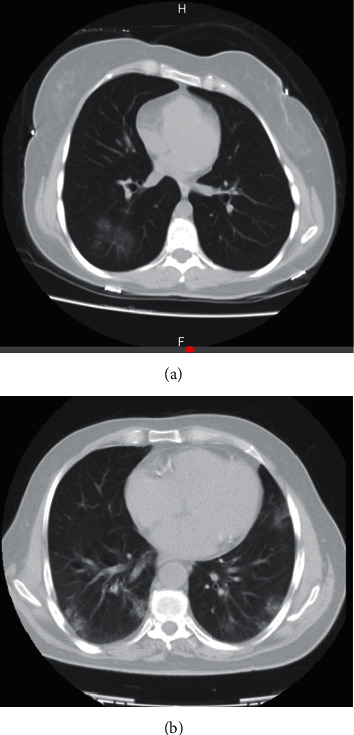
(a) Ground-glass opacities in two different cases of COVID-19 in an early stage. (b) Ground-glass opacities in two different cases of COVID-19 in an early stage.

In a systematic review on imaging findings of 919 patients (after excluding duplicated cases), Salehi et al. described that “Known features of COVID-19 on initial CT include bilateral multilobar ground-glass opacification (GGO) with a peripheral or posterior distribution, mainly in the lower lobes and less frequently within the right middle lobe” [8]. Likewise, bilateral GGO or patchy opacity as high as 90% was reported in these cases [12] (Figures 1(a) and 1(b)).

Although atypical imaging features such as consolidative opacities superimposed on GGO, septal thickening, bronchiectasis, pleural thickening, and subpleural involvement are less common than subpleural GGO, they are not uncommon [13]. As reported in Zhao et al.' study, more than 50% of patients show the following findings: mixed GGO and consolidation (64.4%), vascular enlargement within lesions (71.3%), and traction bronchiectasis (52.5%) [3].

One hundred and forty-nine RT-PCR confirmed positive patients in a retrospective study were included; 17 patients had normal CT on admission, and 12 cases (8%) had negative CT findings even 10 days later [14].

On the other hand, pleural effusion, pericardial effusion, lymphadenopathy, cavitation, CT halo sign, and pneumothorax were very rarely reported. The increasing size and number of GGO transforming to multifocal consolidative opacities, septal thickening (with or without the nodular pattern), and the development of a crazy-paving pattern are important markers of progression. Also, in contrast, gradual resolution of consolidative opacities and decrease in the number of lesions and involved lobes are useful as improvement markers for response to the treatment [8].

Several radiologic signs frequently reported in bacterial pneumonia are reported in some studies [15]. For example, Zhou et al. reported vacuolar sign (45.2%), microvascular dilation sign (56.5%), fibrotic streaks (33.9%), a subpleural line (53.2%), air bronchogram (72.6%), pleural thickening (48.4%), and a pleural retraction sign (56.5%) [16].

Although patchy, peripheral, and multifocal GGO opacities are frequently reported as the main COVID-19 imaging findings, some studies have reported other findings and even normal CT scans in these patients. For example, Chung et al. distinguished that 14% of cases have no GGO or consolidation and had entirely normal chest CT examinations at presentation [17]. No CT or radiographic abnormality was found in about 18% of cases with nonsevere disease and 2.9% of cases with severe disease in Guan's study. Wu's study found that 31.25% of cases had a normal CT of both lungs, and finally, 23% of cases had a normal bilateral chest CT in the study by Xu et al. [6, 18, 19].

It is not always the case that all patients first develop GGO and later develop consolidation or other imaging features, since recently published studies report that some atypical manifestations can be seen in certain cases (e.g., elderly or pediatric cases). These unusual presentations will be discussed in the following section. The distribution of affected lobes is different in previous studies, but the posterior aspect of the lower lobes is often initially involved [15, 20].

**Figure 2:**
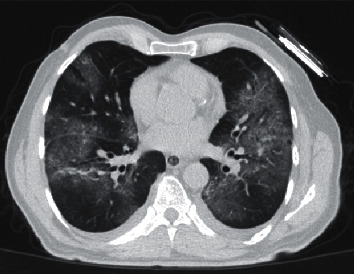
GGO with interlobular septal thickening in a case of COVID-19 at the subacute phase (note to pleural effusion).

Finally, recent insights provided by Ye et al. describe that ground-glass opacities, consolidation, a reticular pattern, and a crazy-paving pattern are typical CT manifestations of COVID-19, and in contrast, airway changes, pleural changes, fibrosis, and nodules are atypical CT manifestations [21] (Figure 2).

## 4. Review of Atypical Radiologic Findings

In spite of GGOs and patchy opacities as usual findings of COVID-19 pneumonia in unenhanced chest CT, Lei et al. reported the absence of sparing of the subpleural regions in contrast to previous studies [22]. Moreover, Lin et al. published a case report of an asymptomatic case of COVID-19 pneumonia with bilateral pleural effusions, which was not previously reported [23].

Albarello et al. reported 2 cases with moderate to severe progression of lung infiltrates that included, in addition to pleural effusions, a tubular and enlarged appearance of pulmonary vessels with a sudden caliber reduction, mainly found in the dichotomic tracts, where the center of a new insurgent pulmonary lesion could be seen. Furthermore, mediastinal lymphadenopathy with short-axis oval nodes was also reported in a deteriorated case. They suggested these findings as early alert radiological signs to predict initial lung deterioration in COVID-19 cases [24].

Another study stated that, in addition to peripheral lower lobe GGOs as a routine CT manifestation, vascular thickening with a pleural parallel sign (the most valuable characteristic), intralobular septal thickening, pleural effusion, pneumatocele, pneumothorax, and less commonly halo/reversed halo sign were also seen in these cases [25, 26].

### 4.1. Radiologist Performance in Diagnosis of COVID-19

In addition to recognizing and diagnosing imaging findings in these patients, the radiologist should assist the clinicians in the following areas: diagnosis of underlying pulmonary abnormalities, evaluation of the severity and extent of the disease regarding architectural distortion, traction bronchiectasis, CT involvement score [3], interpretation of positive chest CT findings in some cases with negative results of PCR, especially during the first five days [27], avoiding unnecessary CT scans in patients with low probability for COVID-19 as a screening modality, distinguish unusual CT presentations from other causes according to clinical and laboratory findings, severity score determination of pulmonary involvement to distinguish critical cases from others [15], diagnosis of the complications of COVID-19, and protect radiology departments and its personnel from infections.

Severity score and classification of the disease should be performed, although its effectiveness and usefulness are not sufficiently studied. Chung et al. used a lung severity score in COVID-19 cases according to the following: “Each of the five lung lobes was assessed for degree of involvement and classified as none (0%), minimal (1%–25%), mild (26%–50%), moderate (51%–75%), or severe (76%–100%). No involvement corresponded to a lobe score of 0, minimal involvement to a lobe score of 1, mild involvement to a lobe score of 2, moderate involvement to a lobe score of 3, and severe involvement to a lobe score of 4. An overall lung total severity score was reached by summing the five lobe scores (range of possible scores, 0–20)” [17].

Based on previous evaluations, it can be concluded that patients with the following findings are more likely to progress to severe stages: architectural distortion, traction bronchiectasis, and a higher CT involvement score [3]. Also, round cystic changes might be associated with the resolving process of consolidation or could be explained by the infection causing alveolar wall damage, leading to pneumatoceles [28].

Using serial CT studies to determinate outcome and prognosis, radiologists can contribute to prognostication. Shi et al. described an evolution of three pattern types of CT findings, in which type 2 has a poor prognosis. If CT findings showed initial progression to a peak level, followed by radiographic improvement, the pattern type is type 1. A pattern of radiographic improvement across several CT scans is categorized as type 3, and progressive radiographic deterioration despite medical treatment was categorized as type 2 with poor prognosis [28].

### 4.2. Radiologic Findings in Time Course and Follow-Up

CT findings typically peak within 10 days of the onset of symptoms and subsequently decrease from two to five weeks, depending on the patient's clinical condition and underlying disorders. Almost always in usual cases, there are GGOs at early evaluation and then an increase in the size and number of GGOs. This progression of GGOs to multifocal consolidation, crazy-paving pattern formation, and septal thickening continues until the 10–14th day of diseases (peak of radiologic findings). Then, there is gradual resolution of consolidative opacities and a decrease in the number of involved lobes and lesions seen by 4-5 weeks.

Salehi et al. described that septal thickening, pleural thickening, bronchiectasis (usually at the later phase), pleural effusion, lymphadenopathy, pericardial effusion, CT halo sign, cavitation, and pneumothorax are seen during the progressive and complicating phase [8].

CT studies of 63 cases, with a mean age of about 50 years, show that the mean number of affected lobes on admission was more than 3 lobes. Progression from single GGO to enlarged and consolidated opacities, enlarged fibrous stripe, and increased and enlarged solid nodules were also described [29].

Bernheim et al. reviewed chest CT findings of 121 symptomatic COVID-19 cases based on the time between symptom onset and the initial CT scan. They categorized cases into 3 groups, including early (0–2 days), intermediate (3–5 days), and late (6–12 days). A normal CT was found in more than half of the cases in the early stage. With a longer time after the onset of symptoms, CT findings were more frequent, including consolidation, bilateral and peripheral diseases, greater total lung involvement, linear opacities, a crazy-paving pattern, and the reverse halo sign. Bilateral lung involvement was observed in 28% of patients in the early phase, 76% of patients in the intermediate phase, and 88% of patients in the late phase [30].

Although the development of bilateral GGOs and consolidation is frequently reported, solitary and rounded peripheral ground-glass lesions appearing 3 days after the follow-up was also reported [17].

**Figure 3:**
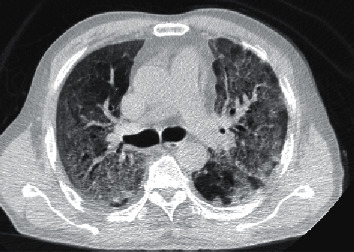
Diffuse consolidation in a case at the peak phase resembling ARDS.

Pan et al. postulated that based on the day of symptom onset, 4 stages of lung CT  could be defined as follows: stage 1 (0–4 days), ground-glass opacities (GGO) in 75% of patients; stage 2 (5-8 days), increased the crazy-paving pattern in 53%; stage 3 (9–13 days), consolidation in 91%; and stage 4 ( ≥ 14 days), gradual resolution of consolidation in 75% of patients without the crazy-paving pattern [31]. Peak lung involvement was approximately on the 10th day with a mean hospitalized period of 17 days (Figure 3). The cohort excluded ARDS and hypoxemic cases, and their results are generalizable for cases with mild and moderate severity. More than the extension and distribution of GGO, it appears that the densities of GGO indicate disease progression [32]. Clinically, in addition to the densities of GGO seen on CT, monitoring of hypoxemia is a valuable indicator of severity [33].

The following findings were obtained in a study of 81 cases who were categorized based on symptom onset and the first CT scan. In group 1 (subclinical patients; scans done before symptom onset), the predominant pattern was unilateral and multifocal GGO. Lesions quickly evolved to bilateral, diffuse GGO in group 2 (scans done ≤ 1 week after symptom onset). Thereafter, the prevalence of ground-glass opacities continued to decrease in group 3 (1 to 2 weeks) and group 4 (2 to 3 weeks), and consolidation and mixed patterns became more frequent in these aforementioned groups [28].

GGOs opacities change to several subtypes during follow-up. Wang et al. demonstrated that at first, on illness days 6–11, pure GGO was the most common subtype of GGO, with rates of up to 71%, followed by GGO with irregular linear opacity (28%). The most common pattern was mixed GGO in 38% of cases until days 12–17. About 94% of cases discharged had residual manifestations on final CT scans, with GGO in 60% of cases and pure GGO in up to 74% of cases being the most common pattern and subtype [34].

In spite of all the above featured reports, it should be noted that normal CT scan findings have been frequently reported in these patients so that, in a study on 149 RT-PCR confirmed positive patients, 17 (11%) patients had normal CT on admission and 12 (8%) had negative findings even 10 days later [14].

Early CT Manifestations of 108 patients with COVID-19 pneumonia are “patchy GGO with or without consolidation involving multiple lobes, mainly in the peripheral zone, accompanied by halo sign, vascular thickening, the crazy-paving pattern, or air bronchogram sign,” which is partially different from other reports that demonstrated that the above findings, except for patchy GGOs, can be seen in the early stages and the remainder in the more advanced stages [35]. The difference can be due to inclusion criteria of cases (only mild COVID-19 pneumonia is included), and there is no definite time of symptom onset and the first CT scan obtained.

CT studies of 63 confirmed patients show that 85% of patients had progressed in their disease with the following findings: increasing single GGO, enlarged and consolidated opacities, enlarging fibrous stripe, and increasing and enlarging solid nodules [29].

Zhu et al. reported CT  scan findings of 50 cases. On first imaging and in the early phase, GGOs were found in 92.3% of cases, patchy consolidation and subconsolidation in 36.5%, and air bronchogram in 32.7% of cases. During hospitalization, the fibrous stripe shadow became the most usual imaging findings (75.0% within 6–9 days after admission), and the lesions distinctly resolved in 76% of cases on days 10–14 of admission [36].

**Figure 4:**
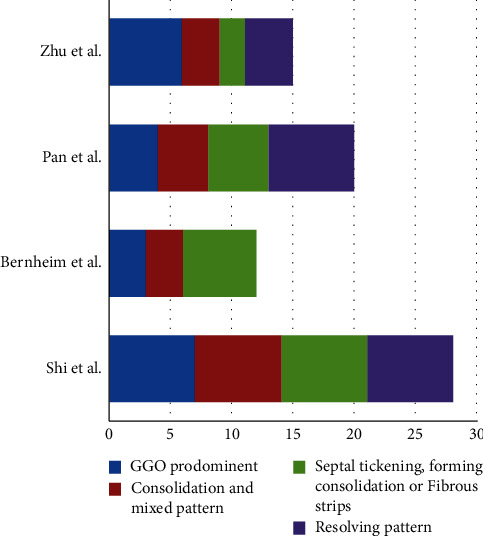
Comparison of CT findings in four studies with time course follow-up.

The schematic review of the time course of COVID-19 CT findings is illustrated in Figure 4.

### 4.3. Radiologic Findings in Unusual Cases

The radiologic findings reported in the preceding sections are mainly those most commonly observed, while different radiological findings have been reported in patients with specific conditions. For example, multiple lobe involvement is more commonly reported in the elderly [37]. On the other hand, Zhu et al. reported that the ratio of central and gradient distribution of GGOs, thickening of interlobular septum, and rounded morphology were higher in cases with heart failure rather than usual cases. The ratio of central and gradient distribution of the enlargement of small pulmonary veins was also higher in heart failure cases. As expected, this enlargement disappeared after the administration of antiheart failure medication [38].

COVID-19 has also been reported in pregnant patients with a radiologic profile similar to that of other patients [27]. However, differences have also been reported. Lie et al. demonstrated that GGO and reticulation were less common in the pregnant groups (6% and 5%) versus that of nonpregnant adults (18%). Therewith, mixed consolidation and complete consolidation were more common in pregnant groups (more than 40%) compared with 28%of the nonpregnant adults [39].

### 4.4. Radiologic Findings in Children vs. Adults

The disease is less symptomatic in children and is less severe, but the radiological findings reported in the pediatric age group are relatively similar to those of adults [40]. However, there are slight differences [39].

**Figure 5:**
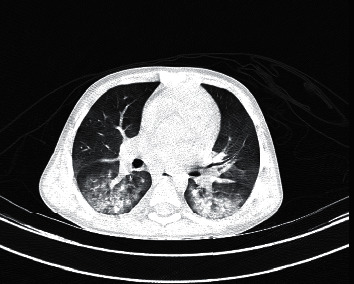
A 9-year-old case with the proven COVID-19 PCR test and bilateral lower lobes GGO.

After assessment of chest CTs of 20 pediatrics cases of COVID-19, Xie et al. reported consolidation with surrounding halo sign (50%), GGOs (60%), fine mesh shadow (20%), and tiny nodules (15%), and 20% cases showed no abnormality (Figure 5). Consolidation with surrounding halo signs (suggesting underlying coinfection) was common in children with COVID-19 and unlikely to be present in adults [27]. In contrast to adults cases, pleural effusion and a “white lung-like” change is also reported in as much as 10% cases [41].

Therefore, in children with suspected symptoms and clinical history, mild and sometimes unusual CT findings should be considered.

### 4.5. Radiologic Findings and Correlated Clinical/Paraclinical Findings

We et al. showed that a “pulmonary inflammation index (PII)” was positively correlated with the lymphocyte count, monocyte count, procalcitonin level, CRP (C-reactive protein), days of illness onset, and body temperature. These laboratory markers have been previously reported as a prognostic factor of COVID-19 in some studies. The PII, which Wu et al. used, is according to the guideline of Chongqing Radiologist Association of China and is based on distribution and severity of involved lung segments. In this scoring system, each involved lung segment is scored (with a max score of 20, which means all lobes are involved). If a lesion occupies more than 50% of a lung segment, it receives a score of 1, and if the lesion occupies less than 50%, it receives a score of 0 [42]. This scoring system can be used by the radiologist as a predictive factor of symptomatic cases, although further studies are needed to determine its value as an indicator of prognosis.

Some laboratory factors were reported to correlate with radiologic findings in these cases. The CRP, erythrocyte sedimentation rate (ESR), and lactate dehydrogenase (LDH) had a significant positive correlation with the severity of pneumonia in the first CT, and higher temperature and the severity of opacification in the first CT were considerably correlated with the progression of opacification on the follow-up CT [43]. The sensitivity of CRP, ESR, and decreased WBC was reported as high as 100%, 67%, and 80% of COVID-19 cases [16].

Reactivated cases with different laboratory findings and similar radiologic findings are reported more often than usual laboratory findings of COVID-19 cases; Ye et al. reported 5 cases of COVID-19 reactivation with typical signs of a viral infection on CT scan, but one case had progressive lymphopenia and progressive neutrophilia, and all of them had normal aminotransferase levels [44]. Just as there is a significant difference between imaging findings of young/middle age patients and the elderly, there are also different findings in the laboratory values. Liu et al. described that “The proportion of lymphocytes in the elderly group was significantly lower than that in the young and middle-aged groups, and the CRP was significantly higher in the young group” [37].

The RT-PCR false negative rate is considerable in COVID-19 and has been reported to be up to 50%. In cases with highly suspicious history of exposure and clinical and laboratory findings, chest CT was recommended. Likewise, Ai et al. stated that “the sensitivity of chest CT in suggesting COVID-19 was 97% based on positive RT-PCR results. By analysis of serial RT-PCR assays and CT scans, the mean interval time between the initial negative to positive RT-PCR results was more than 5 days.” In the follow-up chest CT scans, 42% of patients showed improvement before the RT-PCR results returned as negative [45].

Besides RT-PCR, other laboratory data versus CT findings have shown acceptable sensitivity. Xu et al. reported 100% negative chest CTs in mild cases of COVID-19 with more than 50% of cases having increased CRP and 28% of cases showing changes in the WBC count [46].

In addition to the CT  severity index as a predicting factor of deterioration, some laboratory findings, which include higher WBC and PMN counts, higher levels of D-dimer, creatine kinase, and creatine, are different between patients admitted to the ICU compared to those who were not admitted. All cases in both groups, however, showed bilateral lung involvement in chest CT scans. The median time from symptom onset to admission to the ICU was 10 days [47].

In addition to using chest CT to distinguish COVID-19 cases from non-COVID-19 cases, abnormal laboratory tests in AST, ALT, *γ*-GT, LDH, and *α*-HBDH can also be useful [27].

Laboratory findings will be helpful in pediatric cases, which have less obvious clinical symptoms and radiological findings when compared to adult cases. In pediatric cases, consolidation with surrounding halo signs and increased procalciton were more common when compared to adults, although pediatric patients are more susceptible to coinfection compared to adults [27].

### 4.6. Radiologic Findings Based on Symptoms and Severity of the Disease

Most studies review the radiologic manifestation of COVID-19 in symptomatic cases, but asymptomatic cases also have a specific radiologic pattern. Knowing this pattern can help diagnose suspicious individuals. Fifty percentage (50%) of asymptomatic carriers with a positive PCR test showed typical CT findings of ground-glass opacity, 20% presented with stripe shadowing in the lungs, and only 29% of cases showed a normal CT study [48]. Of note, many studies report that CT scanning provides positive results sooner than PCR [49, 50].

**Figure 6:**
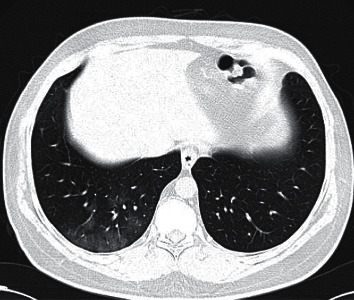
Beginning of GGO at the subclinical phase with nonfrequent cough.

**Figure 7:**
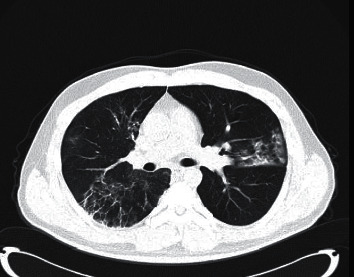
A middle age man after treatment and at 12^th^ day of symptoms onset with fibrotic changes.

Similar to the most previous studies, Pan et al. reported that “mild COVID-19 pneumonia mainly starts as small subpleural, unilateral or bilateral GGO in the lower lobes” [31] (Figure 6). These lesions develop into subsequent consolidation and a crazy-paving pattern. The residual GGO and subpleural parenchymal bands appear gradually after two weeks, indicating a decrease in the severity of the disease (Figure 7). This engenders the question whether radiological findings can distinguish mild cases from severe disease in need of intensive care. In other words, what are the findings in computed tomography of severe predictive of intensive care transfer? We will discuss this further.

Chung et al. reported that the cases with the highest lung severity score at HRCT were admitted to the intensive care unit [17].

Data from 101 cases of COVID-19 pneumonia show that patients in the emergency group are more likely to have the following findings: architectural distortion, traction bronchiectasis, and higher CT involvement score [3].

Bilateral patchy consolidation and interstitial abnormalities on CT and CXR are reported to occur twice as frequently as in nonsevere patients [6]. This finding is generally expected in all pulmonary diseases with alveolar infections due to progression of the disease in critical cases.

A report of 62 patients with COVID-19 pneumonia comparing CT findings of the early phase (≤7 days after the onset of symptoms) to the advanced phase (8–14 days after the onset of symptoms) showed considerably increased prevalence of GGO plus a reticular pattern, fibrotic streaks, vacuolar sign, a subpleural transparent line, a subpleural line, bronchus distortion, air bronchogram, and pleural effusion. Meanwhile, GGOs diminished dramatically in advanced stages. These findings show a mixed pattern with both the interstitium and lung parenchyma patterns in the late stage [16].

The incidences of lymph node enlargement, pericardial effusion, and pleural effusion were higher in critical cases [3]. Li et al. described that “consolidation, linear opacities, crazy-paving pattern, bronchial wall thickening, high CT scores, and extrapulmonary lesions were features of severe/critical COVID-19 pneumonia” [9].

In children with critical status, similar chest CT findings were reported, but decreased CD16 + CD56 and Th/Ts, increased CD3, CD4, and CD8, IL-6 and IL-10, and increased IFN-*γ* were also reported [41].

### 4.7. Radiologic Findings and Outcomes

Predominantly diffused consolidations associated with ARDS are described as radiological findings in near-death studies of patients who eventually expired. ARDS is characterized as the final pathogenesis in severe pneumonia of COVID-19, and ARDS was reported in as many as 29% of admitted cases [51]. Radiologic findings in these cases are similar to ordinary ARDS [52, 53].

Fibrous tissue formation, which includes increased septal thickness and fibrotic stripes, is seen in improved patients, patients in the recovering phase, and in patients in the progressive stages, confirming significant parenchymal damage [43, 54]. Two mechanisms can explain the pathogenesis of these fibrotic fibers: underlying inflammatory processes and cytokine induced injuries and the external pressure from a ventilator, which expands the alveolar space, filling it with exudative material [55].

Further studies with comparing groups of ventilated versus nonventilated cases are recommended.

Although many patients have partial improvement in symptoms within two weeks, many do not respond to routine treatment and require intensive care. Lei et al. demonstrated that almost 54% of cases are refractory patients with male sex, anorexia, and no fever on admission, which predicted poor outcome [22]. This high rate of refractory patients may be due to the limited capacity of hospitals in epidemic conditions, where only patients with severe illnesses and the majority of COVID-19 pneumonia patients receive outpatient care. Finally, it seems that elderly patients with underlying diseases and severe radiological changes are most likely to fail to respond to treatment and progress to more severe stages [56].

Li et al. explained that “CT findings of consolidation, linear opacities, crazy-paving pattern, bronchial wall thickening, high CT  scores, and extrapulmonary lesions were features of severe/critical COVID-19 pneumonia” [9]. These results partially contrast the prognostic radiological findings reported in patients with MERS (pleural effusion and higher CT lung and chest radiographic scores) [57]. This is the difference of being involved in the pathogenesis of the two diseases (MERS vs.SARS-CoV-2).

### 4.8. Radiologic Findings in COVID-19 and Non-COVID-19

A study on 19 cases with COVID-19 pneumonia and 15 cases with other types of pneumonia demonstrated that clinical symptoms were similar in these two groups. However, “78.95% of COVID-19 and 26.67% of non-NCOVID-19 patients had bilateral involvement, while 17 (89.47%) COVID-19 and 1 (6.67%) non-NCOVID-19 patients had multiple mottling and GGOs on chest CT images” [27]. Another similar study described that GGO (91% vs. 68%), peripheral distribution (80% vs. 57%), vascular thickening (59% vs. 22%), fine reticular opacity (56% vs. 22%), and reverse halo sign (11% vs. 1%) were significantly more common in COVID-19 cases compared to non-COVID-19 cases. Whereas air bronchogram (14% vs. 23%, *p* = 0.014), lymphadenopathy (2.7% vs. 10.2%), pleural thickening (15% vs. 33%), and pleural effusion (4 vs. 39%) are less common in COVID-19 cases [58].

Li and Xia reported similar findings in comparison of pneumonia in COVID-19 versus adenovirus cases, but more studies may be needed since their findings are based on a control group with a small sample size [59].

Predominant patterns of lung abnormalities in SARS cases include GGO (with or without superimposed linear opacities), consolidation, a reticular pattern, and a mixed pattern (consolidation, GGO, and a reticular pattern). During the 1st week, GGO with or without smooth interlobular septal thickening and dense opacities were the outstanding patterns. “GGO with superimposed irregular reticular opacities, a mixed pattern, and reticular opacities were noted from the 2nd week and peaked at or after the 4th week.” After the 4th week, more than half of all cases had irregular linear opacities, with or without associated GGOs, and bronchial dilatation [60]. The aforementioned findings are partially similar to COVID-19 findings.

The frequency of chest CT findings in patients with positive results versus patients with negative results (non-COVID-19 pneumonia) was as follows: GGO, 100.0% vs. 90.9%; mixed GGO, 63.6% vs. 72.7%; consolidation, 54.5% vs. 77.3%; the median number of affected lung lobes, 5 vs. 3.5; and affected segments, 15 vs. 9. In patients with positive PCR, the air bronchogram reticular pattern was more frequent, but centrilobular nodules were uncommon [61].

Patchy and GGO opacities in middle and lower lobes with bronchial wall thickening and the nodular pattern have been confirmed to be more common in viral pneumonias. Nodularity is not frequent in these patients, and up to 40% of patients have the nodular pattern [62]. Also, halo appearance and round lung opacities can be seen in up to 33% of the chest CTs of symptomatic patients with COVID-19 [56]. Therefore, the above routine viral presentations inchest CT of COVID-19 are not expected.

## 5. Review on Biopsy Pathologic Findings

Diffuse alveolar damage as the main histopathologic pattern was found in pneumonia due to MERS. Type 2 pneumocytes and epithelial syncytial cells are the predominant target of viral antigens. Cytopathic effects of MERS contribute to respiratory symptoms. Detachment of type 2 pneumocytes and membrane blebbing (suggestive of apoptosis in favor of pneumocyte damage, in addition to other causes such as immune dysfunction) may be involved in the pathogenesis [63].

An autopsy report noted desquamation of pneumocytes, hyaline membrane formation, and pulmonary edema with hyaline membrane formation, indicating ARDS. “Interstitial mononuclear inflammatory infiltrates dominated by lymphocytes were seen in both lungs.” Viral cytopathic-like changes without obvious intranuclear or intracytoplasmic viral inclusions were noted [64].

These findings are not very useful because they were performed in deceased patients that had died in the severe phase of respiratory failure of ARDS. Therefore, it is expected that routine findings of ARDS, like in other non-COVID-19 ARDS, were present.

However, in a unique study, Tian et al. reported pathologic findings of two cases of incidental COVID-19. The two cases underwent biopsy for their malignancy and were later found to have been infected with SARS-CoV-2. Because these specimens were collected in the early phase of the disease, these findings show a more realistic view of the pathological findings than the autopsy samples. They obtained the following findings in both cases: “edema and prominent proteinaceous exudates, vascular congestion, and inflammatory clusters with fibrinoid material and multinucleated giant cells.” The findings noted reactive alveolar epithelial hyperplasia in one case and fibroblastic proliferation in another. No prominent neutrophil infiltration or large protein globules were reported. The aforementioned findings are usual in patients with SARS [65]. During this earlier phase, these observations are in favor of early organization and are seen in many acute inflammatory viral/nonviral injuries. Further studies regarding the progressive/intermediated phase are mandatory to assess the immune host response as a therapeutic target.

## 6. Correlation with Radiology Findings and Pathogenesis

GGO is defined as “hazy increased attenuation of the lung, but with preservation of bronchial and vascular margins, caused by partial filling of air spaces, interstitial thickening, partial collapse of alveoli, normal expiration, or increased capillary blood volume” [66]. Based on CT scans, 4 types of GGO may be recognized: type I as simple ground-glass-like shadow, type II as uneven density, type III as central high density with peripheral burring, and type IV as nodular GGO [67]. GGO lesions present as incomplete filling of the alveolar cavity with fluid or lung interstitial thickening. As expected, in COVID-19 cases, patchy GGO is noted due to alveolar cell injuries and inflammatory processes. However, this inflammation is not limited to pneumocytes, and depending on the immune response and severity of the inflammatory process, interstitial tissue, vessels, bronchioles, and even the pleural layer may be involved.

GGO with prominent vessels has been previously reported for tumorous GGO like bronchoalveolar adenocarcinoma, but many reports of COVID-19 reported these findings frequently, especially in periods of disease progression [68].

Vascular leaking and increased permeability in response to increased cytokines can be seen in early lung injuries (with GGO) and in the myocardial injuries also reported in COVID-19. In the progressive phase of the development of ARDS, “damage of epithelial cells and release of proteases from neutrophils decreases the VEGF level in the alveolar compartment, while serum VEGF is elevated” [69].

Patchy GGO was previously reported in the case of interstitial pneumonia, which is “characterized by a mononuclear inflammatory cellular infiltrate in the alveolar septa and the distal peribronchovascular interstitium. This interstitial inflammatory reaction is secondary to epithelial damage, with thickening in the peribronchial area and interlobular septa. The most common causes are viruses, *M. pneumoniae*, and *P. jirovecii.*” Some cases develop into acute necrotic pneumonia, which overlaps with ARDS due to severe injuries of alveolar cells [70]. In the early stages of injury, many laboratory and imaging findings are consistent with an acute infectious pulmonary injury focused on the alveoli; however, the healing process in many of these patients is different than in almost all lung infections. This progressive process is more consistent with organizing pneumonia (OP).

**Figure 8:**
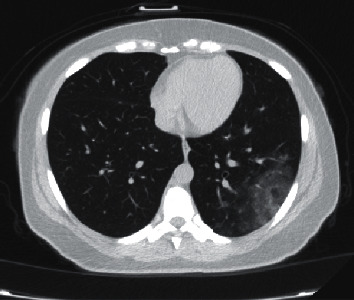
Halo sign in a case of PCR proved COVID-19.

In addition to GGOs in peripheral lower lobes as a routine CT manifestation, vascular thickening with pleural parallel sign (the most valuable characteristic), intralobular septal thickening, pleural effusion, and pneumatocele, and less commonly, halo/reversed halo sign were also seen in these cases (Figure 8). In the later period, it mainly manifested as organizing pneumonia and fibrosis [25]. In the study by Qin et al., 18F-FDG PET/CT was used for COVID-19 patients who had peripheral GGO and/or lung consolidations in more than two pulmonary lobes. “Lung lesions were characterized by a high 18F-FDG uptake, and there was evidence of lymph node involvement. Conversely, disseminated disease was absent, a finding suggesting that COVID-19 has pulmonary tropism.” This finding is consistent with the hyperemic situation in this disease [71]. Therefore, assessing how VEGF and other cytokines affect vessel walls based on CT  manifestations may be a target of further study.

Furthermore, Huang et al. commented on the role of interleukin- (IL-) 2, IL-7, granulocyte-colony stimulating factor, interferon-*γ*-inducible protein 10, monocyte chemoattractant protein 1, macrophage inflammatory protein 1-*α*, and tumor necrosis factor-*α* in ICU admitted cases of COVID-19 versus non-ICU cases [51]. These mediators have a role in immune system balance in pneumonia. There is an important balance between the inflammatory cytokines and the immunomedulatory cytokines. If either become abnormally elevated, secondary lung injury can occur due to the immune system hyperactivation or the progression of the infection [72]. This dysregulation of the immune system response is more frequent in the elderly, which is the population most at risk of critical stages of COVID-19 [73]. Likewise, the severity of disease in children with the undeveloped acquired immune system is more subtle.

This hyperinflammatory condition may be in line with secondary haemophagocytic lymphohistiocytosis (sHLH), which is most often triggered by viral infections. This syndrome is fulminant, and fatal hypercytokinaemia with multiorgan failure can result, which has been previously reported as one of the possible mechanisms of pathogenesis of the disease. Increased ferritin, decreased platelet counts, elevated procalcitonin, CRP, and ESR in the severe stage of COVID-19 are also consistent with this syndrome, and for this reason, steroids, IVIG, specific cytokine blockade (e.g., baricitinib, anakinra, or tacilizumab), and JAK inhibition can be effective for these patients [74, 75].

In addition to GGO, mosaic attenuation patterns are a recognized finding in some viral infections, which was uncommonly reported in COVID-19. Hypoventilation of alveoli distal to bronchiolar obstruction (cicatricial scarring or inflammation of many bronchioles), which leads to secondary vasoconstriction (and, consequently, underperfused lung) is the main mechanism of mosaic attenuation patterns. This is different than the possible mechanism of COVID-19. This mechanism is based on two main pathologies, including vasospasm and narrowing of bronchioles, which seems to be absent in COVID-19. Bronchiolar obstruction and restriction, and therefore bronchiolitis obliterans organizing pneumonia (BOOP), are more common in other viral pneumonia and are not seen in COVID-19. Bronchial wall sparing or minimal involvements in COVID-19 indicate and suggest that treatments targeting small airway will be less effective in these patients [76]. More than OP, acute interstitial pneumonitis (AIP) is another possible pathogenesis of the middle phase of COVID-19. AIP reported as an entity encompasses a wide variety of etiologies that are often unidentified, in which some may be viral in origin [77].

## 7. Conclusion

**Figure 9:**
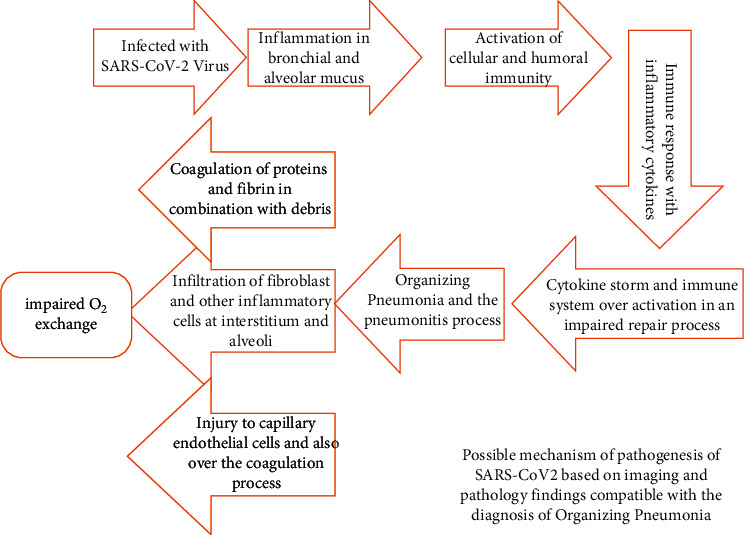
Diagram illustrating the main pathophysiological steps of COVID-19-related lung infection and damage (inflammatory and fibrotic).

In conclusion, our hypothesis states that after the early phase of pneumonia, some cases develop into an inflammatory/fibrotic phase of OP, and cases with underlying disorders develop into the severe form of OP with predominance of the inflammatory/toxic phase (Figure 9) (severe OP). On the other hand, some cases in the early phase present radiologic and laboratory results similar to acute interstitial pneumonitis (mixed condition of alveolar infection and its cytotoxicity and interstitial changes due to host reactive inflammation). We suggest that these cases are more susceptible for progression to the severe phase. The role of the innate immune system may be more prominent in these cases. Development to each phase is dependent on the host's immunologic reaction and response severity. Therefore, some cases had a positive response from corticosteroid and immunomedulatory medications, while it had no effect in other cases. Although imaging is known to be a predictor of outcomes, additional studies on the possible role of radiologic-pathologic correlations in the guidance of therapy may be needed.

## Data Availability

No data were used to support this study.

## Additional Points

We would like to discuss the diagnosis of SARS-COVID-19 and also underlying pulmonary abnormalities; evaluation of the severity and extent of the disease regarding architectural distortion; traction bronchiectasis; CT involvement score; interpretation of positive chest CT findings in some cases with negative results of PCR, especially during the first five days; avoiding unnecessary CT scans in patients with low probability for COVID-19 as a screening modality; distinguish unusual CT presentations from other causes according to clinical and laboratory findings; severity score determination of pulmonary involvement to distinguish critical cases from others; diagnosis of the complications of COVID-19; and the final possible pathogenesis related to the imaging findings.

## References

[1] Young B. E., Ong S. W. X., Kalimuddin S. (2020). Epidemiologic features and clinical course of patients infected with SARS-CoV-2 in Singapore. *JAMA*.

[2] Kanne J. P., Little B. P., Chung J. H., Elicker B. M., Ketai L. H. (2020). Essentials for radiologists on COVID-19: an update—radiology scientific expert panel. *Radiology*.

[3] Zhao W., Zhong Z., Xie X., Yu Q., Liu J. (2020). Relation between chest CT findings and clinical conditions of coronavirus disease (COVID-19) pneumonia: a multicenter study. *American Journal of Roentgenology*.

[4] Caruso D., Zerunian M., Polici M. (2020). Chest CT features of COVID-19 in Rome, Italy. *Radiology*.

[5] Ajlan A. M., Ahyad R. A., Jamjoom L. G., Alharthy A., Madani T. A. (2014). Middle East respiratory syndrome coronavirus (MERS-CoV) infection: chest CT findings. *American Journal of Roentgenology*.

[6] Guan W.-J., Ni Z.-Y., Hu Y. (2020). Clinical characteristics of coronavirus disease 2019 in China. *New England Journal of Medicine*.

[7] Mo P., Xing Y., Xiao Y. (2020). Clinical characteristics of refractory COVID-19 pneumonia in Wuhan, China. *Clinical Infectious Diseases: An Official Publication of The Infectious Diseases Society of America*.

[8] Salehi S., Abedi A., Balakrishnan S., Gholamrezanezhad A. (2020). Coronavirus disease 2019 (COVID-19): a systematic review of imaging findings in 919 patients. *American Journal of Roentgenology*.

[9] Li K., Wu J., Wu F. (2020). The clinical and chest CT features associated with severe and critical COVID-19 pneumonia. *Investigative Radiology*.

[10] Watanabe M., Caruso D., Tuccinardi D. (2020). Visceral fat shows the strongest association with the need of intensive care in patients with COVID-19. *Metabolism*.

[11] Hong H., Wang Y., Chung H.-T., Chen C.-J. (2019). Clinical characteristics of novel coronavirus disease 2019 (COVID-19) in newborns, infants and children. *Pediatrics & Neonatology*.

[12] Zhang J. J., Dong X., Cao Y. Y. (2020). Clinical characteristics of 140 patients infected with SARS-CoV-2 in Wuhan, China. *Allergy*.

[13] Caruso D., Polidori T., Guido G. (2020). Typical and atypical COVID-19 computed tomography findings. *World Journal of Clinical Cases*.

[14] Yang W., Cao Q., Qin L. (2020). Clinical characteristics and imaging manifestations of the 2019 novel coronavirus disease (COVID-19): a multi-center study in Wenzhou city, Zhejiang, China. *Journal of Infection*.

[15] Zu Z. Y., Jiang M. D., Xu P. P. (2020). Coronavirus disease 2019 (COVID-19): a perspective from China.. *Radiology*.

[16] Zhou S., Wang Y., Zhu T., Xia L. (2020). CT features of coronavirus disease 2019 (COVID-19) pneumonia in 62 patients in Wuhan, China. *American Journal of Roentgenology*.

[17] Chung M., Bernheim A., Mei X. (2020). CT imaging features of 2019 novel coronavirus (2019-nCoV). *Radiology*.

[18] Wu J., Liu J., Zhao X. (2020). Clinical characteristics of imported cases of COVID-19 in Jiangsu province: a multicenter descriptive study. *Clinical Infectious Diseases: An Official Publication of the Infectious Diseases Society of America*.

[19] Xu X., Yu C., Qu J. (2020). Imaging and clinical features of patients with 2019 novel coronavirus SARS-CoV-2. *European Journal of Nuclear Medicine and Molecular Imaging*.

[20] Zhang M. Q., Wang X. H., Chen Y. L. (2020). Clinical features of 2019 novel coronavirus pneumonia in the early stage from a fever clinic in Beijing. *Zhonghua Jie He He Hu Xi Za Zhi*.

[21] Ye Z., Zhang Y., Wang Y., Huang Z., Song B. (2020). Chest CT manifestations of new coronavirus disease 2019 (COVID-19): a pictorial review. *European Radiology*.

[22] Lei J., Li J., Li X., Qi X. (2020). CT imaging of the 2019 novel coronavirus (2019-nCoV) pneumonia. *Radiology*.

[23] Lin C., Ding Y., Xie B. (2020). Asymptomatic novel coronavirus pneumonia patient outside Wuhan: the value of CT images in the course of the disease. *Clinical Imaging*.

[24] Albarello F., Pianura E., Di Stefano F. (2020). 2019-novel Coronavirus severe adult respiratory distress syndrome in two cases in Italy: an uncommon radiological presentation. *International Journal of Infectious Diseases*.

[25] Wu J., Feng C. L., Xian X. Y. (2020). Novel coronavirus pneumonia (COVID-19) CT distribution and sign features. *Zhonghua Jie He He Hu Xi Za Zhi*.

[26] Chen N., Zhou M., Dong X. (2020). Epidemiological and clinical characteristics of 99 cases of 2019 novel coronavirus pneumonia in Wuhan, China: a descriptive study. *The Lancet*.

[27] Xie X., Zhong Z., Zhao W., Zheng C., Wang F., Liu J. (2020). Chest CT for typical 2019-nCoV pneumonia: relationship to negative RT-PCR testing. *Radiology*.

[28] Shi H., Han X., Jiang N., Cao Y., Alwalid O., Gu J. (2020). Radiological findings from 81 patients with COVID-19 pneumonia in Wuhan, China: a descriptive study. *The Lancet Infectious Diseases*.

[29] Pan Y., Guan H., Zhou S. (2020). Initial CT findings and temporal changes in patients with the novel coronavirus pneumonia (2019-nCoV): a study of 63 patients in Wuhan, China. *European Radiology*.

[30] Bernheim A., Mei X., Huang M. (2020). Chest CT findings in coronavirus disease-19 (COVID-19): relationship to duration of infection. *Radiology*.

[31] Pan F., Ye T., Sun P. (2020). Time course of lung changes on chest CT during recovery from 2019 novel coronavirus (COVID-19) pneumonia. *Radiology*.

[32] Fang Y., Zhang H., Xie J. (2020). Sensitivity of chest CT for COVID-19: comparison to RT-PCR. *Radiology*.

[33] Rello J., Tejada S., Userovici C., Arvaniti K., Pugin J., Waterer G. (2020). Coronavirus disease 2019 (COVID-19): a critical care perspective beyond China. *Anaesthesia, Critical Care & Pain Medicine*.

[34] Wang Y., Dong C., Hu Y. (2020). Temporal changes of CT findings in 90 patients with COVID-19 pneumonia: a longitudinal study. *Radiology*.

[35] Han R., Huang L., Jiang H., Dong J., Peng H., Zhang D. (2020). Early clinical and CT manifestations of coronavirus disease 2019 (COVID-19) pneumonia. *American Journal of Roentgenology*.

[36] Zhu Y., Liu Y.-L., Li Z.-P. Clinical and CT imaging features of 2019 novel coronavirus disease (COVID-19). *Journal of Infection*.

[37] Liu K., Chen Y., Lin R., Han K. (2020). Clinical feature of COVID-19 in elderly patients: a comparison with young and middle-aged patients. *Journal of Infection [Review]*.

[38] Zhu Z. W., Tang J. J., Chai X. P. (2020). Comparison of heart failure and 2019 novel coronavirus pneumonia in chest CT features and clinical characteristics. *Zhonghua Xin Xue Guan Bing Za Zhi*.

[39] Liu H., Liu F., Li J., Zhang T., Wang D., Lan W. (2020). Clinical and CT imaging features of the COVID-19 pneumonia: focus on pregnant women and children. *Journal of Infection*.

[40] Li W., Cui H., Li K., Fang Y., Li S. (2020). Chest computed tomography in children with COVID-19 respiratory infection. *Pediatric Radiology*.

[41] Sun D., Li H., Lu X.-X. (2020). Clinical features of severe pediatric patients with coronavirus disease 2019 in Wuhan: a single center’s observational study. *World Journal of Pediatrics*.

[42] Wu J., Wu X., Zeng W. (2020). Chest CT findings in patients with corona virus disease 2019 and its relationship with clinical features. *Investigative Radiology*.

[43] Xiong Y., Sun D., Liu Y. (2020). Clinical and high-resolution CT features of the COVID-19 infection: comparison of the initial and follow-up changes. *Investigative Radiology*.

[44] Ye G., Pan Z., Pan Y. (2020). Clinical characteristics of severe acute respiratory syndrome coronavirus 2 reactivation. *Journal of Infection*.

[45] Ai T., Yang Z., Hou H. (2020). Correlation of chest CT and RT-PCR testing in coronavirus disease 2019 (COVID-19) in China: a report of 1014 cases. *Radiology*.

[46] Xu Y. H., Dong J. H., An W. M. (2020). Clinical and computed tomographic imaging features of novel coronavirus pneumonia caused by SARS-CoV-2. *Journal of Infection*.

[47] Wang D., Hu B., Hu C. (2020). Clinical characteristics of 138 hospitalized patients with 2019 novel coronavirus-infected pneumonia in Wuhan, China. *JAMA*.

[48] Hu Z., Song C., Xu C. (2020). Clinical characteristics of 24 asymptomatic infections with COVID-19 screened among close contacts in Nanjing, China. *Science China Life sciences*.

[49] An P., Song P., Lian K., Wang Y. (2020). CT manifestations of novel coronavirus pneumonia: a case report. *Balkan Medical Journal*.

[50] Zhu W., Xie K., Lu H., Xu L., Zhou S., Fang S. (2020). Initial clinical features of suspected coronavirus disease 2019 in two emergency departments outside of Hubei, China. *Journal of Medical Virology*.

[51] Huang C., Wang Y., Li X. (2020). Clinical features of patients infected with 2019 novel coronavirus in Wuhan, China. *The Lancet*.

[52] Yang X., Yu Y., Xu J. (2020). Clinical course and outcomes of critically ill patients with SARS-CoV-2 pneumonia in Wuhan, China: a single-centered, retrospective, observational study. *Lancet Respiratory Medicine*.

[53] Wu C., Chen X., Cai Y. (2020). Risk factors associated with acute respiratory distress syndrome and death in patients with coronavirus disease 2019 pneumonia in Wuhan, China. *JAMA Internal Medicine*.

[54] Wei J., Xu H., Xiong J. (2019). Novel coronavirus (COVID-19) pneumonia: serial computed tomography findings. *Korean Journal of Radiology. [Case Reports]*.

[55] Faffe D. S., Zin W. A. (2009). Lung parenchymal mechanics in health and disease. *Physiological Reviews*.

[56] Kay F., Abbara S. (2020). The many faces of COVID-19: spectrum of imaging manifestations. *Radiology: Cardiothoracic Imaging*.

[57] Das K. M., Lee E. Y., Enani M. A. (2015). CT correlation with outcomes in 15 patients with acute Middle East respiratory syndrome coronavirus. *American Journal of Roentgenology*.

[58] Bai H. X., Hsieh B., Xiong Z. Performance of radiologists in differentiating COVID-19 from viral pneumonia on chest CT. *Radiology*.

[59] Li Y., Xia L. (2020). Coronavirus disease 2019 (COVID-19): role of chest CT in diagnosis and management. *American Journal of Roentgenology*.

[60] Ooi G. C., Khong P. L., Müller N. L. (2004). Severe acute respiratory syndrome: temporal lung changes at thin-section CT in 30 patients. *Radiology*.

[61] Cheng Z., Lu Y., Cao Q. (2020). Clinical features and chest CT manifestations of coronavirus disease 2019 (COVID-19) in a single-center study in shanghai, China. *American Journal of Roentgenology*.

[62] Yoon S. H., Lee K. H., Kim J. Y. (2020). Chest radiographic and CT findings of the 2019 novel coronavirus disease (COVID-19): analysis of nine patients treated in Korea. *Korean Journal of Radiology*.

[63] Ng D. L., Al Hosani F., Keating M. K. (2016). Clinicopathologic, immunohistochemical, and ultrastructural findings of a fatal case of Middle East respiratory syndrome coronavirus infection in the United Arab Emirates. *The American Journal of Pathology*.

[64] Xu Z., Shi L., Wang Y. (2020). Pathological findings of COVID-19 associated with acute respiratory distress syndrome. *Lancet Respiratory Medicine [Case Reports]*.

[65] Tian S., Hu W., Niu L., Liu H., Xu H., Xiao S.-Y. (2019). Pulmonary pathology of early-phase 2019 novel coronavirus (COVID-19) pneumonia in two patients with lung cancer. *Journal of Thoracic Oncology*.

[66] Austin J. H., Müller N. L., Friedman P. J. (1996). Glossary of terms for CT of the lungs: recommendations of the nomenclature committee of the fleischner society. *Radiology*.

[67] Yu H., Liu S., Zhang C. (2018). Computed tomography and pathology evaluation of lung ground-glass opacity. *Experimental and Therapeutic Medicine*.

[68] Liu G., Li M., Li G. (2018). Assessing the blood supply status of the focal ground-glass opacity in lungs using spectral computed tomography. *Korean Journal of Radiology*.

[69] Papp Á., Bene Z., Gáspár I. (2015). Decreased VEGF level is associated with elevated ferritin concentration in bronchoalveolar lavage fluid of children with interstitial lung diseases. *Respiration*.

[70] Beigelman-Aubry C., Godet C., Caumes E. (2012). Lung infections: the radiologist’s perspective. *Diagnostic and Interventional Imaging*.

[71] Qin C., Liu F., Yen T. C., Lan X. (2020). (18) F-FDG PET/CT findings of COVID-19: a series of four highly suspected cases. *European Journal of Nuclear Medicine and Molecular Imaging*.

[72] Kerr A. R., Irvine J. J., Search J. J. (2002). Role of inflammatory mediators in resistance and susceptibility to pneumococcal infection. *Infection and Immunity*.

[73] Boyd A. R., Orihuela C. J. (2011). Dysregulated inflammation as a risk factor for pneumonia in the elderly. *Aging and Disease*.

[74] Mehta P., McAuley D. F., Brown M., Sanchez E., Tattersall R. S., Manson J. J. (2020).

[75] Richardson P., Griffin I., Tucker C. (2020). Baricitinib as potential treatment for 2019-nCoV acute respiratory disease. *The Lancet*.

[76] Franquet T. (2011). Imaging of pulmonary viral pneumonia. *Radiology*.

[77] Galante O., Avni Y. S., Fuchs L., Ferster O. A., Almog Y. (2016). Coronavirus NL63-induced adult respiratory distress syndrome. *American Journal of Respiratory and Critical Care Medicine*.

